# Renal Transplant Outcomes in Plasma Cell Dyscrasias and AL Amyloidosis after Treatment with Daratumumab

**DOI:** 10.3390/jcm13144109

**Published:** 2024-07-14

**Authors:** Barian Mohidin, Amy Needleman, Raymond Fernando, David M. Lowe, Ashutosh Wechalekar, Michael Sheaff, Alan Salama, Gareth Jones

**Affiliations:** 1 UCL Department of Renal Medicine, Royal Free Hospital, London NW3 2QG, UK; 2 H&I Laboratory, Royal Free Hospital, London NW3 2QG, UK; 3 UCL Institute of Immunity & Transplantation, Royal Free Hospital, London NW3 2QG, UK; 4 Department of Haematology, Royal Free Hospital, London NW3 2QG, UK; 5 Department of Cellular Pathology, Royal London Hospital, London NW3 2QG, UK

**Keywords:** plasma cell dyscrasia, AL amyloidosis, renal transplantation, daratumumab, immunoglobulin

## Abstract

**Background:** The morbidity and mortality from AL amyloidosis has significantly improved with the development of novel treatments. Daratumumab is a highly effective treatment for AL amyloidosis, but end-stage kidney disease is a common complication of this condition. Kidney transplantation is the ideal form of renal replacement therapy but has historically been contraindicated in this group of patients. **Methods:** Given the improved survival and better treatments of both conditions, we argue that it is time to reconsider transplanting these patients. **Results:** We report our experience of transplanting four patients with AL amyloidosis who had achieved stable remission through treatment with daratumumab. **Conclusions:** We highlight the key challenges involved and discuss important clinical issues for patients receiving daratumumab, particularly the difficulties with interpreting the crossmatch in light of daratumumab and immunoglobulin therapy interference. We also discuss the complexities involved in balancing the risks of infection, relapse, rejection, and immunosuppression in such patients.

## 1. Introduction

AL amyloidosis results from clonal plasma cells producing excessive light chains which misfold into fibrils and deposit in extracellular tissue causing organ injury [1]. The incidence is 1.2 cases per 100,000 people per year [2]. The kidneys are the organs most often involved at 74% [3]. Progression to end-stage kidney disease is common and the prospect of renal recovery with treatment is rare [4]. However, with the advent of new treatments, prognosis has significantly improved, and five-year survival has increased to 80% [5].

Given the historically poor survival rates, inability to cure the disease, and high risk of relapse, patients with AL amyloidosis have not been considered for kidney transplantation [2]. However, novel therapeutic agents have produced a dramatic improvement in survival so patients with AL amyloidosis should be reconsidered for kidney transplantation.

Daratumumab is a human IgG1 kappa monoclonal antibody that binds to CD38 [6]. It is a potent inhibitor of CD38 expressing cells through a variety of mechanisms, including antibody-dependent cellular cytotoxicity, antibody-dependent phagocytosis, and complement-dependent cytotoxicity [7,8]. CD38 is a transmembrane glycoprotein expressed by plasma cells, plasmablasts, natural killer cells, and regulatory B- and T-lymphocytes [9]. Daratumumab will therefore have an effect on immune cells other than plasma cells and the duration of action on these cells is unknown, although it has a half-life between 9 and 27 days [10].

The use of daratumumab has dramatically improved the outcomes in AL amyloidosis. Clinical trials show that the addition of daratumumab to standard of care chemotherapy in AL amyloidosis increases complete haematological response rates to 33% and improves progression-free survival compared to standard of care chemotherapy [11]. We present our experience, through this case series, of transplanting patients who had achieved complete or near-complete response through the use of daratumumab.

## 2. Materials and Methods

Case records of patients with AL amyloidosis secondary to plasma cell dyscrasias who had achieved remission through use of daratumumab and had subsequently received a kidney transplant were reviewed and their experience described below.

## 3. Results

### 3.1. Case One

**Figure 1:**
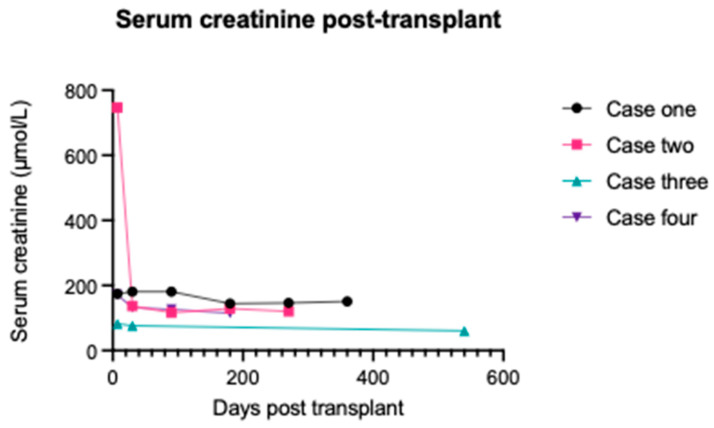
Serum creatinine post-transplant.

**Figure 2:**
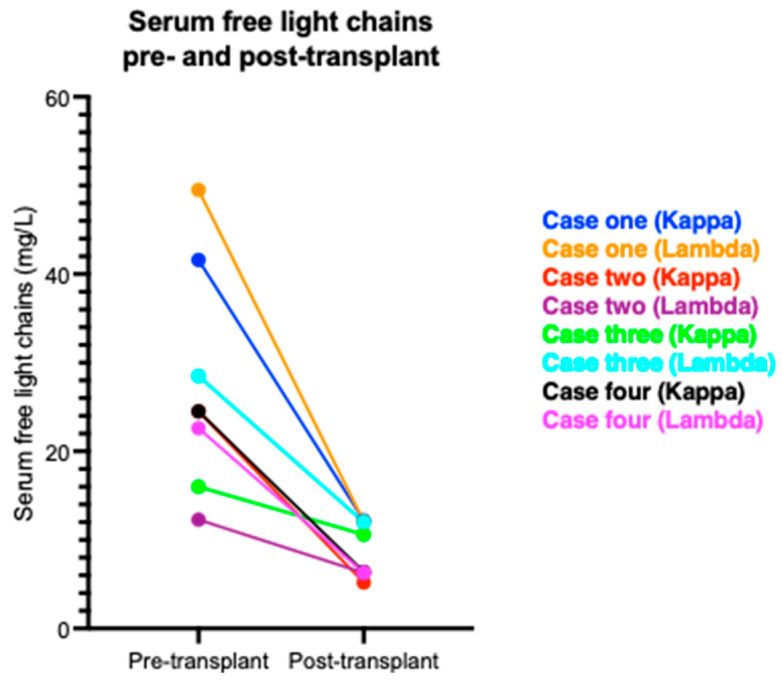
Serum free light chains pre- and post-transplant.

A 45-year-old male was diagnosed with a lambda light chain-secreting plasma cell dyscrasia and AL amyloidosis in February 2018. He achieved a partial haematological response (more than 50% reduction in the difference in free light chains) with bortezomib, lenalidomide, and dexamethasone combination chemotherapy. Consequently, he started daratumumab after which he achieved a complete haematological response (normal free light chain ratio with no detectable paraprotein). Nevertheless, he remained grossly nephrotic from his AL amyloidosis, and he commenced haemodialysis in February 2021 following an admission with COVID-19. Profound secondary hypogammaglobulinaemia was evident with a serum immunoglobulin G (IgG) level of 2.5 g/L. However, monthly intravenous immunoglobulin infusions were delayed until post-transplantation, given his lack of recurrent bacterial infections at this time. He received a living-related donor kidney transplant from his brother in September 2022. The HLA mismatch was 0-1-1 at the A, B, and DR loci, respectively. He had no anti-HLA donor specific antibodies (DSA). The autologous (auto) and allogeneic (allo) B-cell IgG flow cytometry crossmatches were negative. However, the allo- T-cell IgG flow cytometry crossmatch was positive whereas the auto- was negative. The blood group crossmatch was also positive. However, no blood group alloantibodies were identified with dithiothreitol-treated cells on the indirect antiglobulin test. Primary function was achieved post-transplantation. Basiliximab was used for induction immunosuppression and tacrolimus and mycophenolate mofetil for maintenance immunosuppression, following a rapid steroid wean. His creatinine was 144 µmol/L (1.63 mg/dL) and 151 µmol/L (1.71 mg/dL) at 6 and 12 months, respectively (Figure 1). During this period, he has remained in remission from his plasma cell dyscrasia and AL amyloidosis (Figure 2). He has not suffered any episodes of biopsy-proven acute rejection or developed any anti-HLA DSAs. Despite regular immunoglobulin therapy, he has suffered four infectious complications: superficial wound infection, influenza-positive respiratory tract infection, asymptomatic cytomegalovirus (CMV) viraemia (donor IgG negative, recipient IgG positive), and extended spectrum beta-lactamase producing Klebsiella pneumoniae urinary tract infection. However, he did not require hospitalisation for any of these infections. He has not required any further daratumumab since his transplant; however, a recent bone marrow biopsy has revealed low-level minimal residual disease, and he is being considered for an autologous stem cell transplant.

### 3.2. Case Two

**Figure 3:**
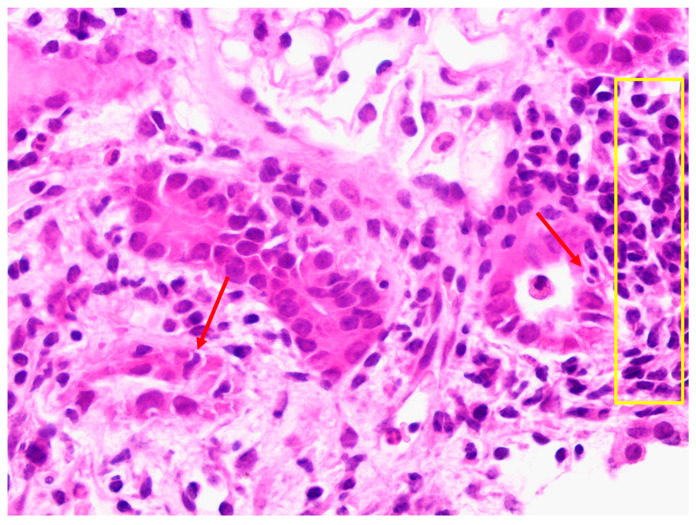
Allograft biopsy from case two (haematoxylin and eosin stain) showing borderline T-cell-mediated rejection. An interstitial infiltrate is seen (yellow rectangle) as well as a degree of tubulitis (red arrows).

A 59-year-old male was diagnosed with an IgG kappa-secreting plasma cell dyscrasia and AL amyloidosis in August 2019 and commenced haemodialysis for end-stage kidney disease. He achieved a partial haematological response with bortezomib, cyclophosphamide, and dexamethasone combination chemotherapy, but this was complicated by the development of a severe peripheral neuropathy. He relapsed in June 2020 and commenced daratumumab after which he achieved a complete haematological response. IgG levels were low pre-transplant at 2.5 g/L. He received a 41-year-old female deceased kidney donor (donation after brainstem death) in January 2023. The HLA mismatch was 2-2-2 at the A, B, and DR loci, respectively. He had no anti-HLA DSAs, and the transplant proceeded on the basis of a negative virtual crossmatch. The retrospective crossmatch performed using T- and B-lymphocytes isolated from donor spleen was allo- T- and B-cell IgG flow cytometry crossmatch positive whereas the auto- crossmatches were negative. No anti-HLA DSAs were identified on the day of transplant using Luminex HLA antibody specificity identification kits. The blood group crossmatch was also positive. However, no alloantibodies were identified with dithiothreitol-treated cells on the indirect antiglobulin test. Basiliximab was used for induction immunosuppression and tacrolimus and mycophenolate mofetil for maintenance immunosuppression, following a rapid steroid wean. He experienced delayed graft function postoperatively and required haemodialysis for hyperkalaemia. A mercaptoacetyltriglycine renogram suggested resolving acute tubular necrosis, but a day 8 biopsy revealed features in keeping with borderline rejection and significant acute tubular injury (Figure 3). His serum creatinine significantly improved without treatment, and he was discharged. His creatinine at 6 months was 129 µmol/L (1.46 mg/dL) and his current creatinine at 9 months is 120 µmol/L (1.36 mg/dL) (Figure 1). He has not developed any anti-HLA DSAs to date. Monthly intravenous immunoglobulin infusions were commenced for secondary hypogammaglobulinaemia post-transplantation. During this period, he has remained in remission from his plasma cell dyscrasia and AL amyloidosis (Figure 2). He restarted maintenance daratumumab in April 2023 but suffered asymptomatic CMV viraemia (donor IgG positive, recipient IgG positive) and BK viraemia after restarting daratumumab, which required a dose reduction in his maintenance immunosuppression, a short course of valganciclovir, and a brief interruption to his daratumumab therapy schedule. He has not required hospitalisation for any of these infections or other issues post transplantation.

### 3.3. Case Three

A 61-year-old female was diagnosed with lambda light chain-secreting plasma cell dyscrasia and AL amyloidosis in September 2021. She commenced haemodialysis shortly after her diagnosis. No cardiac involvement was seen on magnetic resonance imaging. She achieved a partial haematological response with bortezomib, cyclophosphamide, and dexamethasone combination chemotherapy. Daratumumab was administered as a second-line therapy in November 2021 after which she achieved a complete haematological response. She received a living-related donor kidney transplant from her son in April 2022. The HLA mismatch was 1-1-0 at the A, B, and DR loci, respectively. She had no anti-HLA DSAs. The auto- and allo- B-cell IgG flow cytometry crossmatches were negative. However, the allo- T-cell IgG flow cytometry crossmatch was positive whereas the auto- was negative. The blood group crossmatch was also positive. However, no alloantibodies were identified with dithiothreitol-treated cells on the indirect antiglobulin test. Primary function was achieved post-transplantation. Basiliximab was used for induction immunosuppression and tacrolimus and mycophenolate mofetil for maintenance immunosuppression, following a rapid steroid wean. Her creatinine at 1 month was 76 µmol/L (0.86 mg/dL) (Figure 1). She returned to her country of origin after 1 month and was lost to follow-up. During the first postoperative month, she remained in remission from her plasma cell dyscrasia and AL amyloidosis (Figure 2), did not suffer any episodes of biopsy-proven acute rejection, nor did she develop any anti-HLA DSAs. She had one infectious complication in the form of asymptomatic CMV viraemia (donor IgG positive, recipient IgG positive) but did not require hospitalisation. However, she had one hospital admission associated with symptomatic second-degree atrioventricular heart block with a 2:1 pattern. A pacemaker was fitted. Her creatinine at 18 months is 60 µmol/L (0.68 mg/dL).

### 3.4. Case Four

A 62-year-old female with a lambda light chain-secreting plasma cell dyscrasia was diagnosed with AL amyloidosis in 2016. She commenced peritoneal dialysis shortly thereafter before transferring to haemodialysis in 2018. Her plasma cell dyscrasia was treated with six cycles of bortezomib, cyclophosphamide, and dexamethasone combination chemotherapy and she achieved a complete haematological response. She relapsed in 2018 and was treated with a cycle of cyclophosphamide, thalidomide, and dexamethasone, but she experienced multiple side effects. The combination was switched to lenalidomide, ixazomib, and dexamethasone until February 2019, when her regime was changed again to daratumumab and dexamethasone, after which she achieved a complete haematological response. Subcutaneous weekly immunoglobulin therapy was commenced in September 2022 for secondary hypogammaglobulinaemia and a poor response to polysaccharide pneumococcal vaccination. Following cardiac work up, she received a deceased donor kidney transplant from a 70-year-old female (donation after brainstem death) in May 2023. She was sensitised with a calculated reaction frequency (cRF) of 93%. The HLA mismatch was 2-1-0 at the A, B, and DR loci, respectively. She had no anti-HLA DSAs. Her pre-transplant allo- and auto- T-cell and B-cell IgG flow cytometry crossmatches were negative. A retrospective crossmatch using spleen lymphocytes also confirmed the negative allogeneic crossmatches. However, the blood group crossmatch was positive, but no alloantibodies were identified with dithiothreitol-treated cells on the indirect antiglobulin test. Basiliximab was used for induction immunosuppression and tacrolimus and mycophenolate mofetil for maintenance immunosuppression, following a rapid steroid wean. Her postoperative course was uncomplicated, and she achieved primary function. Her creatinine at 6 months is 115 µmol/L (1.3 mg/dL). She continues to receive monthly maintenance daratumumab and remains in remission. She also takes subcutaneous immunoglobulin weekly and has experienced one infectious complication post-transplantation in the form of a streptococcus gallolyticus urinary tract infection. She has not had any hospital admissions post-transplantation.

## 4. Discussion

Prognosis for patients with AL amyloidosis on dialysis is inferior to patients on dialysis from other causes [12,13]. Historically, there has been a reluctance to consider transplantation in such cases due to poor outcomes with early graft loss, disease recurrence, and shorter overall survival [14,15].

Our case series shows that it is possible to transplant patients with good 6-month outcomes, but we await their long-term results. Reviewing the literature, outcomes of renal transplantation in patients with AL amyloidosis has traditionally been poor [14]. However, with significant advancement in clone-directed therapy, haematological outcomes have significantly improved and so have renal transplant outcomes. Angel-Korman et al. noted significant improvement in both patient and graft survival since 2007 [16], which coincides with the introduction of bortezomib. They followed 49 individuals post-renal transplantation with AL amyloid for a median of 7 years and found a 5-year overall survival of 86% and a graft survival of 81% [16]. Median survival was 12 years, compared to a predicted survival of 2–3 years had they not been transplanted [16]. No individual received daratumumab pre-transplant, but the majority (80%) had undergone a stem cell transplant. Twelve patients (24%) were treated with both conventional chemotherapy (including bortezomib) and stem cell transplantation. One third (29%) experienced recurrence with a median onset at 3 years. Acute rejection was only seen in four patients. The best predictor of survival was a haematological complete response or very good partial response.

The numbers being transplanted have also increased with each decade. Havasi et al. reported 20 patients receiving a renal transplant before 2000, 91 between 2000 and 2010, and 128 between 2011 and 2020 [17]. Havasi et al. also reported on the outcomes of 237 patients with AL amyloid who received kidney transplants in different countries between 1987 and 2020 [17]. The median follow-up was 8 years, and they reported a five-year overall and graft survival of 74% and 69%, respectively. The median graft survival was 7 years, and the best outcomes (both patient and graft survival) were seen in patients who achieved a complete haematological response or a very good partial response pre-transplant. One third experienced amyloid recurrence with the median time to recurrence being 6 years, but only 13% of this group experienced graft failure. T-cell-mediated rejection was seen in 13% and antibody-mediated rejection in 3%. Post-transplant lymphoproliferative disease was diagnosed in five patients. Interestingly, they found no difference in outcomes between patients who underwent stem cell transplant and those treated with chemotherapy only. Moreover, there was no difference in survival between living and deceased donation.

Heybeli et al. reported an overall survival median of 10 years in 60 patients transplanted with AL amyloid. Their death-censored graft survival was 96% at 5 years, and 22% experienced recurrence [18]. Law et al. followed-up 51 patients with AL amyloidosis who received a renal transplant for a median of 6 years [19]. They reported a 5-year overall survival of 84% although two died in the first 3 months post-transplant. None received daratumumab pre-transplant. Recurrence was seen in 14% with a median of 4.5 years post-transplant, but only one suffered graft failure because of it. The strongest predictor of survival was, once again, complete haematological response, and the strongest predictor of mortality was echocardiographic findings of an intraventricular septum thickness greater than 12 mm.

None of the series highlighted above demonstrated rejection rates to be higher than typical rates seen in the current era of immunosuppression. However, it is worth noting that rejection rates were not lower either, despite the total burden of immunosuppression they receive (both clone-directed therapy and to prevent allograft sensitisation). There has been concern that daratumumab may reduce CD38-expressing regulatory T- and B-cells, which could potentially result in increased T-cell-mediated rejection [9]. Scalzo et al. report an individual who suffered acute T-cell-mediated rejection within 48 h of transplantation [10]. He had aggressive multiple myeloma and only managed remission after an autologous bone marrow transplant and monthly maintenance daratumumab infusions. He was unsensitised with no HLA antibodies and received a mismatched kidney from his sister in the context of a negative T-cell and B-cell flow cytometry crossmatch. A for-cause biopsy due to rising creatinine levels on day 4 showed features consistent with Banff IIb acute T-cell-mediated rejection, which responded to further immunosuppressive treatment. The rapid onset of rejection suggests the presence of activated circulating T-cells, and in an otherwise immunologically low-risk patient, daratumumab was thought to be an instigating trigger (last received two weeks prior to transplant). Krejcik et al. showed that after daratumumab administration, CD4- and CD8-expressing T-cells increased both in the circulation and bone marrow. Viola et al. found that daratumumab increased natural killer cell activation and T-cell co-stimulation through the CD28-CD80/86 pathway [20]. Interestingly, in our cohort, two patients had a CD8 lymphocytosis but all had a CD19 lymphopenia.

Kwun et al. demonstrated that daratumumab administration reduced DSA levels in sensitised primates pre-transplant [21]. Graft survival was longer in primates who received daratumumab; however, all four rhesus macaques who received daratumumab experienced a rebound in DSA as well as T-cell-mediated rejection. Plasma regulatory B- and T-cells reduced in number whilst activated T-cells increased post-daratumumab infusion. Konstantin et al. reported on an individual who developed myeloma 13 years after receiving a kidney transplant [22]. Coincidentally, he also suffered chronic antibody-mediated rejection. He was treated with daratumumab for myeloma and the investigators noticed an incidental improvement in DSA numbers, improvement in biopsy features of chronic antibody-mediated rejection, and an improvement in his serum creatinine. However, protocol biopsies revealed new subclinical borderline T-cell-mediated rejection, which they treated with methylprednisolone. Interestingly, his regulatory B- and T-cell numbers remained stable throughout this period. Furthermore, there are now case reports of use of daratumumab to treat antibody-mediated rejection [22,23].

Our case series shows infectious complications to be common in this group of patients; however, none required hospitalisation. Those treated with daratumumab are at higher risk of infection due to hypogammaglobinaemia, which often persists beyond treatment. Hypogammaglobulinaemia predisposes to a higher rate of infections in kidney transplant recipients including gram-negative sepsis, acute pyelonephritis, and CMV [24]. Despite some controversy, the consensus is that intravenous immunoglobulin (IVIG) administration can reduce this risk by restoring immunoglobulins levels [25,26]. IVIG has also been reported to reduce infections, particularly viral infections, such as CMV and parvovirus [27]. This does not extend to urinary tract infections where the innate immune response is vital [28]. The patients reported in our case series were all hypogammaglobulinaemic and received immunoglobulin therapy post-transplantation (except one who received it pre-transplantation and another who was lost to follow-up) to reduce the risk of infection during the period of maximal immunosuppression post-transplant. It would be important to study the efficacy of IVIG in mitigating infectious risk. It is also important to note that recurrent infections pre-transplant were uncommon despite profound hypogammaglobulinaemia, B-cell lymphopenia (albeit with preserved switched memory B-cell percentage), and impaired polysaccharide vaccine responses. The cause of the hypogammaglobulinaemia in some instances was multifactorial including chemotherapy, daratumumab and nephrotic syndrome.

Daratumumab has the potential to interfere with the outcome of both the blood group and HLA crossmatch. Indeed, we found positive pre-transplantation blood group crossmatches and positive B-cell and T-cell IgG flow cytometry crossmatches in our patients. The combination of positive crossmatches from daratumumab combined with reduced immunoglobulin levels for the detection of HLA antibodies made pre-transplant immunological risk assessment very challenging. Additionally, case four received immunoglobulin therapy pre-transplant which contributed to her high cRF, further complicating the immunological risk assessment. We would advocate a close working relationship between clinicians and colleagues who work in tissue typing and blood transfusion.

CD38 is expressed on red blood cells so circulating daratumumab in the recipient may bind to donor red blood cells [29]. Once Coombs antibodies are added, agglutination will occur and the indirect antiglobulin test will read positive, even without the presence of recipient antibodies directed to donor blood group antigens. A positive blood group crossmatch usually occurs in ABO-incompatible solid organ transplantation, which has a dedicated desensitisation protocol. To investigate whether the positive result was due to ABO-incompatibility rather than daratumumab interference, we repeated the indirect antiglobulin test after mixing with dithiothreitol [30]. This compound disrupts the disulfide bonds in the extracellular domain of CD38 and therefore can overcome any daratumumab interference in the indirect antiglobulin test [29]. Indeed, we were successful using this protocol in our patients with a positive blood group crossmatch, and we were able to achieve negative indirect antiglobulin tests after dithiothreitol addition. However, dithiothreitol also denatures a number of minor blood group antigens including Kell, Dombrock, Indian, and Landsteiner–Wiener antigens. This could potentially be clinically relevant if the recipient has alloantibodies against these minor blood group antigens as the dithiothreitol-treated crossmatch would be rendered negative [30]. This could potentially increase the risk of mild delayed haemolytic transfusion reactions post-blood transfusion in kidney transplant recipients who have received daratumumab.

CD38 is variably expressed on both B- and T-lymphocytes and so circulating daratumumab in the recipient may bind to donor B- and T-lymphocytes [31]. This can give rise to positive HLA crossmatches (both allo- and auto-, B- and T-cell, IgG flow cytometry crossmatches). Indeed, our patients had positive HLA crossmatches against donor lymphocytes, despite lacking anti-HLA DSAs. One limitation of our study is that we did not test for non-HLA antibodies so we cannot comment on whether they may have contributed to the positive HLA crossmatch. Interestingly, although we saw positive results against allo- B-cell and T-cell IgG flow cytometry crossmatches, all auto- B-cell and T-cell IgG flow cytometry crossmatches were negative. One may expect the auto- to also be positive as recipient B- and T-cell lymphocytes also express CD38 (expression not limited to donor lymphocytes). We hypothesise the negative auto- HLA crossmatch to be due to a lack of CD38-expressing recipient B- and T-cells, due to the efficacy of daratumumab in inducing apoptosis in this subgroup of immune cells. This peculiarity was also observed by other investigators [31].

None of our patients had anti-HLA DSAs that we could detect using Luminex single-bead antigen testing. If they did have anti-HLA DSAs, the HLA crossmatch interpretation would be far more challenging as we would need to tease apart whether any positivity was due to HLA incompatibility versus daratumumab interference. Dithiothreitol has been used by one group of investigators to resolve this issue. They report intact HLA antigens after dithiothreitol treatment and no impact on HLA expression or the crossmatch result when anti-HLA DSAs are present [31]. We have also recently validated a method to mitigate the effect of daratumumab interference in our lymphocyte crossmatches [32,33].

## 5. Conclusions

We have presented our experience in transplanting four patients with AL amyloidosis, who have failed first line chemotherapy and achieved a stable remission after daratumumab administration. Their follow-up period ranges from 6 to 18 months. They have achieved excellent graft function with a median creatinine level of 118 µmol/L (1.33 mg/dL). To date, there have been no episodes of biopsy-proven acute rejection, and there have been no clinically significant haematological relapses. Although infectious complications have arisen, none have resulted in hospital admission, and the low sample numbers make it difficult to extrapolate whether the frequency of infectious complications is greater than what would be expected in a matched kidney transplant recipient cohort that did not suffer with AL amyloidosis. It is possible that concurrent immunoglobulin therapy may have prevented more infectious complications than were seen. Overall, we feel we have seen good short-term outcomes, and we are keen to see how they progress long-term. This case series shows that transplantation in patients with AL amyloidosis who have failed first line chemotherapy can have good short-term outcomes.

## Data Availability

The original contributions presented in this study are included in this article, further inquiries can be directed to the corresponding author.
